# Spirit Rappings in the French Academy of Sciences

**Published:** 1854-09

**Authors:** 


### EDITORIAL DEPARTMENT.

*Spirit Rappings in the French Academy of Sciences.*—Very recently this much vexed subject has been brought before the French Academy, by one M. Schiff, and was deemed of sufficient importance to merit discussion in that body—the most distinguished and learned of all scientific societies. This action accords to spiritualism an importance which minds less enlarged are hardly willing to concede. A very few years since, when Dr. Bennett Dowler sent on to the American Medical Association his report as chairman of the Committee on Medical Science, he included Rochester Knockings among the subjects discussed. The reading of this caption was sufficient to cause a shout of derision in this assembly of representative physicians, and so strong a tendency to ridicule was manifested, that, at the request of his colleagues on the committee, this portion of his report was suppressed.

If the American Medical Association were right in their estimate of the importance of the Rochester Knockings, or rather of the results they produce, then, of course, the Academy of Sciences is wrong. But, if we give a moments thought to the subject, we shall see that that is not unimportant which agitates whole communities, destroys religious belief, satisfies the mind with shadows rather than substance, and sends score after score of unfortunate beings to asylums for the insane. If, then, we are able to prove these sounds produced by natural causes, it is certain that a large class of minds will be saved from these deplorable consequences, and though many others may recklessly abandon their reason and better sense to that which they must see to be, at least in part, deception, yet the disaster will not be as wide spread as if no explanation had been offered.

It is remarkable that M. Schiff has arrived at the same conclusion, as to the method of producing the sounds, as that first announced in this city by Prof. Flint, Lee, and Coventry, as the one used by the Fox girls, viz., the partial dislocation of tendons, and their return to the natural position. The peculiar method indicated by M. Schiff was the snapping of the tendon of the peroneus longus over the external malleolus. The Gazette Hebdomadair of July 7th, has a report of the committee to whom the subject was intrusted by the Academy, which accords high praise to M. Schiff for the sagacity and patience of his observations, by which he was not only able to detect the method of their production in a young woman at Frankfort, but finally to produce sounds himself which precisely resemble those ascribed to the spirits.

The identity of motion here proved between this young woman at Frankfort on the Maine and the Fox girls at Buffalo was so striking that Prof. Flint, at that time in Paris, felt justified in calling the attention of the Academy, through M. Rayer, to the fact that the same causes, viz., volition on movable parts of the body, had been reasoned out diagnostically, demonstrated by several cases analogically, and finally proved triumphantly, at a challenged trial with the originators of this rapping system, in the good city of Buffalo, three years ago.

The communication made by Prof. Flint to the Academy of Sciences, through M. Rayer, was submitted by the latter gentleman to M. Robin, who expressed much interest in it, and solicited a copy for the *Societe de Biologie*, of which he is Vice-president.

We have been thus particular in our account of these transactions, for the reason that the true explanation of spirit rapping was first made here by the senior editor of this Journal, associated with Prof's. Lee and Coventry, and at that time occupied considerable space in our pages, and that this explanation, received here with doubt or ridicule, and entailing some obloquy and abuse on its projectors as trivial and insufficient, has, after a lapse of three years, engaged the earnest attention of the highest scientific body in the world, as well as of the Societe de Biologie, (no less distinguished in its peculiar province,) and finally of the scientific journals of Paris, all of which have spoken in complimentary terms of the spirit and logic of Prof. Flint's communication, and received it (with the identical exposè of M. Schiff,) as the true and rational explanation of this most singular delusion.

We subjoin a translation of Prof. Flint's letter read to the Academy of Sciences by M. Rayer, at their sitting of the 3d of July. H.

PARIS, June 28, 1854.

To M. A. RAYER, *Member of the Academy of Sciences, etc., etc.*

SIR:—Having seen in the columns of the *Gazette des Hopitaux* notice of an observation and experiment relative to *spiritual rappers*, by Doctor Schiff, recently communicated by you to the Academy of Sciences, I beg leave to ask of you the favor to transmit to the Academy an account of certain observations and experiments relative to this subject, with which I have been connected.

An exposure of this most remarkable imposition—an imposition which had for years baffled efforts at detection—was published in the United States, in the spring of 1851, by myself, in conjunction with two of my colleagues, viz., Dr. Charles B. Coventry, and Dr. Charles A. Lee. The basis of the exposure was, that the sounds which it was pretended by the authors of the imposition (severel females of a family named Fox) were made by the spirits of the dead, were due to *the voluntary muscles acting on the movable parts of the body.* This principle embraced both movements of the tendons and articulations; and in the course of my investigations, an instance came under my observation precisely similar to that of the young girl examined by Dr. Schiff, and represented by Dr. S. himself before the members of the Academy.

The exposure referred to was published first in a public newspaper at Buffalo, at a time when the Fox girls were in that city holding levees for persons desirous of having intercourse with their deceased relatives. It was shortly afterward communicated to the medical profession in the pages of the Buffalo Medical Journal, of which I am one of the editors; and, subsequently, issued in a brochure. Neither of these printed papers are at hand, at this moment in Paris, but I solicit the favor of transmitting them to the Academy on my return to America. In the mean time, permit me to state the more important of the facts which they contain, which I am able to do perfectly from memory.

The city where I have resided for several years, Buffalo, State of New York, is in the vicinity of the place whence the imposition emanated. The latter is the city of Rochester, and hence the original spiritual rappers are commonly known, in America, as the Rochester rappers.

After visiting several parts of the country, the Fox girls at length honored Buffalo with a visit, and, influenced by curiosity, I was present at one of their exhibitions. A close examination of the countenances of the operators, and the method of conducting the exhibition, satisfied me that the sounds were dependent on the volition of the younger of the two sisters. In discussing the subject afterward, it seemed to me practicable to arrive at the basis of the exposure stated above by the process of reasoning so often useful in diagnosis, viz., reasoning by exclusion. Assuming that the sounds proceeded from volition, it is evident they must be produced either by the voluntary muscles acting on the movable parts of the body, or by mechanical contrivances superadded to the body. That the latter were not employed might be inferred from the fact of the sound being heard under almost every variety of circumstance, and often repeated examinations of the clothing etc., leading to no discovery. Hence, by a logical necessity, the search for the explanation was directed toward the muscles, tendons and articulations, these being the only movable organs under the control of the will.

By a coincidence very remarkable, and which I should attribute to a special interposition of Providence, had the exposure of the imposition sufficed to arrest its progress, a person who was present at the exhibition at the same time as myself, informed me that his wife was able to produce sounds precisely similar to those by means of which the Fox girls were deceiving the public. On visiting this female, I found his statement to be true; and the identity of the sounds as respects character, and intensity, was verified by my colleagues already named, and by several others. This lady permitted us to examine the mode by which she produced the sounds. She was able to effect at will, readily, and without pain, a lateral displacement of the knee-joint. The movement of the bones upon each other gave rise to a loud noise, which, on one occasion, was distinctly heard through a suite of apartments a distance of a hundred feet. She could produce a sound both in moving the bones from their natural position, and in effecting their return; or by causing the displacement or return to take place slowly, she was able to produce a single instead of a double rap. The movements in either direction could be effected silently, and she could graduate the intensity of the sound according to the quickness and force of the movements.

In prosecuting further inquiries, I found another female who possessed an analogous faculty of producing sounds with the knee joint; several instances of ability to produce them by different joints also came under observation, and, as already stated, I met with an instance in which the raps were made by displacement "*in tendon du muscle long pèronier de la gaîne dans laquelle il glisse en passant derrière la malliole externe.*" An account of the instance last mentioned, as well as of the other instances, is given in the publications made by me on this subject in 1851.

These cases were in themselves sufficient to render almost positive the correctness of the principle upon which the exposure by my colleagues and myself was based, viz., that the sounds were due to the voluntary muscles acting on some of the movable parts of the body. But the infatuated assurance of the Rochester girls furnished an opportunity of rendering the demonstration of the correctness of this principle as complete as possible. Fearful of losing the notoriety and pecuniary gains which they derived from the imposition, they were indeed to challenge us publicly, through the papers, to test the truth of our explanation. This challenge was accepted, and the following is a brief account of the interview which took place, by appointment, in the presence of several distinguished gentlemen. Prior to announcing what kind of experiment we should adopt, the spiritual raps were very frequent; and, at our request, the *spirits* were asked if they would consent to hold communication with us, to which an affirmative answer was interpreted by the girls. Acting then, on a hint received from the lady who was able to produce identical sounds, she assuring us that she could not effect the necessary lateral movements unless her feet rested on the floor or against some firm pillar of support, we requested the *rappers* to take a position in the centre of the room on chairs, with the feet elevated and resting on the heels upon soft ottomans. A row of lights was placed on the floor before them in order to detect any sly mode that might be resorted to of violating the conditions of the experiment, and my colleagues and myself took a position for observation in front. Thus arranged, the spirits were asked to fulfil their promise. At our instance they were importuned to adhere to their vow, but no sounds were produced. We waited patiently for nearly an hour, and offered to wait as much longer as was desired, and, indeed, did not consent to relinquish the experiment till the girls themselves declared that they had no expectations of the spirits rapping while they were in that position! Immediately on leaving the chairs and ottomans, and resuming their seats on the sofa, with their feet on the floor, the sounds began to be loud and frequent. On expressing freely our views of the success of the trial, the younger sister began to weep hysterically, and the *seance* was thus terminated.

I wish to add that since my attention was directed to this subject I have met with several persons who were able to produce loud sounds very analogous to the Rochester raps by suddenly and loudly retracting and extending upon the sole of the boot, or shoe, the toes; and it is very probable that the Fox girls, or some of their imitators, may adopt this method, which many, if not all persons may acquire by practice, it not requiring, as do the movements of knee and other joints, a peculiarity of conformation. This method, however involves the same principle, viz., the action of the will on muscles and movable parts. Among the numerous rappers that have sprang up in different portions of the United States, a great variety of means for producing sounds exterior to the body are resorted to, which, however, are generally easily detected. But sounds produced within the organism, by the joints and tendons, were well calculated to elude the discovery of their source, inasmuch as this subject appears hitherto not to have received any scientific examination.

The extent to which spiritual rapping belief has prevailed in the United States offers a sad illustration of the credulity of mankind. Among the ranks of believers are not only the ignorant and persons deficient in mental endowments, but not a few of the educated and persons of high intelligence. A late distinguished incumbent of the bench, and also an ex-governor of one of the territories of the United States are its avowed and ardent advocates. Many persons have adopted opinions and regulated their conduct in most important matters by information which they supposed in this way they received from spirits; and eminent clergymen have sought through this channel for enlightenment as respects the theological tenets which they should receive and inculcate. The results of belief in the imposition have already been very grave. A large number of cases of insanity and suicides occurring in different portions of the country have been fairly attributable to it. It is, therefore certainly a matter of sufficient importance to claim the attention of scientific bodies. This view of the subject, as well as considerations of a personal nature, have led me to desire, to avail myself of the occasion which has now offered itself to present the facts contained in this brief memoir to the Academy of Sciences.

With great respect, yours,

AUSTIN FLINT, M. D.,

Prof. of the Theory and Practice of Med. in the University of Louisville, Kentucky, U. S.

